# Supplementary material for: Length of Washout Period After Remission Does Not Influence Relapse Risk in Patients with Acute Myeloid Leukemia Treated with Hypomethylating Agents Combined with Venetoclax
Source: J Clin Med. 2025 Jul 15;14(14):5007. doi: 10.3390/jcm14145007 (PMC12295650; doi:10.3390/jcm14145007)
Supplement: Supplementary file 1 [file jcm-14-05007-s001.zip › jcm-3708200-supplementary.pdf]

Eligibility criteria according to VIALE-A Trial.

Inclusion Criteria

1. **Diagnosis**: Confirmed acute myeloid leukemia (AML) by WHO criteria, previously untreated.
2. **Age**: ≥18 years.
3. **Life Expectancy**: At least 12 weeks.
4. **Induction Therapy Ineligibility**:
    - **≥75 years old**, OR
    - **18-74 years old** with at least one of the following:
        - ECOG Performance Status of 2 or 3.
        - Cardiac history (CHF requiring treatment, ejection fraction ≤50%, or chronic stable angina).
        - Pulmonary function (DLCO ≤65% or FEV1 ≤65%).
        - Renal impairment (creatinine clearance ≥30 mL/min to <45 mL/min).
        - Moderate hepatic impairment (total bilirubin >1.5 to ≤3.0 × ULN).
        - Other comorbidities deemed incompatible with intensive chemotherapy (subject to approval).
5. **ECOG Performance Status**:
    - 0 to 2 for patients **≥75 years**.
    - 0 to 3 for patients **18-74 years**.
6. **Renal Function**: Creatinine clearance ≥30 mL/min.
7. **Liver Function**:
    - AST and ALT ≤3.0 × ULN.
    - Bilirubin ≤1.5 × ULN (≤3.0 × ULN for patients <75 years).
8. **Female Patients**:
    - Postmenopausal (age >55 with no menses for ≥12 months, or FSH >40 IU/L).
    - Surgically sterile (bilateral oophorectomy, salpingectomy, or hysterectomy).
    - Women of childbearing potential must use protocol-specified contraception.
9. **Male Patients**:

- Must use protocol-specified contraception and refrain from sperm donation for at least 90 days after the last dose.

10. **Pregnancy Testing**:
    - Negative serum pregnancy test within 14 days before first dose.
    - Negative urine pregnancy test on Cycle 1 Day 1 (if serum test was >7 days prior).
11. **Informed Consent**: Signed and dated before screening or study-specific procedures.

Exclusion Criteria

1. **Prior Treatments**:
    - Hypomethylating agents, venetoclax, or chemotherapy for MDS.
    - CAR-T cell therapy.
    - Experimental therapies for MDS or AML.
    - Current participation in another research study.
2. **History of Myeloproliferative Neoplasms**:
    - Myelofibrosis, essential thrombocythemia, polycythemia vera.
    - Chronic myeloid leukemia (CML) with or without BCR-ABL1 translocation.
    - AML with BCR-ABL1 translocation.
3. **Favorable Cytogenetics**:
    - t(8;21), inv(16), t(16;16), or t(15;17) (per NCCN Guidelines).
4. **Specific AML Subtypes**:
    - Acute promyelocytic leukemia.
5. **CNS Involvement**:
    - Known active CNS AML.
6. **Infections**:
    - HIV infection (due to drug interactions).
    - Hepatitis B or C (unless viral load is undetectable within 3 months).
7. **Drug Interactions**:
    - Strong/moderate CYP3A inducers within 7 days before treatment.
    - Consumption of grapefruit, Seville oranges, or starfruit within 3 days before treatment.

8. **Cardiovascular Conditions**:
    - New York Heart Association Class >2.
9. **Respiratory Conditions**:
    - Chronic respiratory disease requiring continuous oxygen.
10. **Other Medical Conditions**:
    - Significant renal, neurologic, psychiatric, endocrinologic, metabolic, immunologic, hepatic, or cardiovascular disease.
    - Known hypersensitivity to study medications.
11. **Malabsorption Syndromes**:
    - Conditions preventing enteral drug administration.
12. **Uncontrolled Systemic Infections**:
    - Viral, bacterial, or fungal infections requiring therapy.
13. **Recent Malignancies**:
    - Within 2 years, except:
        - In situ carcinoma of the cervix or breast.
        - Basal or localized squamous cell carcinoma of the skin.
        - Surgically resected malignancy with curative intent.
14. **High White Blood Cell Count**:
    - WBC $>25 \times 10^9$/L (hydroxyurea or leukapheresis permitted to meet this criterion).
